# Population dynamics of the deer mouse (Peromyscus maniculatus) and Sin Nombre virus, California Channel Islands.

**DOI:** 10.3201/eid0303.970315

**Published:** 1997

**Authors:** T B Graham, B B Chomel

**Affiliations:** USGS, Biological Resources Division, Moab, Utah, USA.

## Abstract

Hantavirus pulmonary syndrome, first documented in 1993, is caused by Sin Nombre virus (SNV), which is carried by the Peromyscus species. In 1994, high SNV antibody prevalence was identified in deer mice from two California Channel Islands. We sampled two locations on three islands to estimate mouse population density and SNV prevalence. Population flux and SNV prevalence appear to vary independently.


### Population Dynamics of the Deer Mouse (*Peromyscus maniculatus*) and Sin Nombre Virus, California Channel Islands

Hantavirus pulmonary syndrome, first documented in 1993, is caused by Sin Nombre virus (SNV), which is carried by the *Peromyscus* species. In 1994, high SNV antibody prevalence was identified in deer mice from two California Channel Islands. We sampled two locations on three islands to estimate mouse population density and SNV prevalence. Population flux and SNV prevalence appear to vary independently.

A new acute respiratory illness, hantavirus pulmonary syndrome (HPS), was first documented in May 1993 in New Mexico (1). The death rate was initially more than 90% (1,2) and is now approximately 50% (3). Serologic surveys indicated positive reactions with previously known hantavirus antigens but not with any agents usually associated with severe respiratory illness (2). Four distinct serotypes of hantaviruses, which are carried by rodents, were known before 1993 (2). The virus causing HPS in the Four Corners area, Sin Nombre virus (SNV), represents an unusual fifth serotype that affects the lungs and has a high death rate (1,4).

During the 1993 outbreak, rodents were trapped in and near homes with confirmed SNV cases and tested for hantavirus antibodies (1). *Peromyscus maniculatus* (deer mouse) was the most common rodent captured and had the highest antibody prevalence. Average prevalence was 30.4% (0% to 51.3% range) for 813 *P. maniculatus* captured at 21 sites (1).

*P. maniculatus* is the only species of mouse on four of the five islands that make up Channel Islands National Park; Santa Cruz Island also has populations of *Reithrodontomys megalotis*. Channel Islands National Park (Figure 1) has been monitoring deer mouse populations on Santa Barbara Island for 19 years (recorded data are incomplete) and on San Miguel and Anacapa Islands since 1993 (5; C. Schwemm, pers. comm.). Concern for the health of persons trapping mice and others on the islands prompted testing of mice on San Miguel, Santa Rosa, and Santa Barbara Islands for hantavirus in 1994. After SNV was identified in mice from these islands, mice from the other five Channel Islands were tested. Blood samples from technicians and

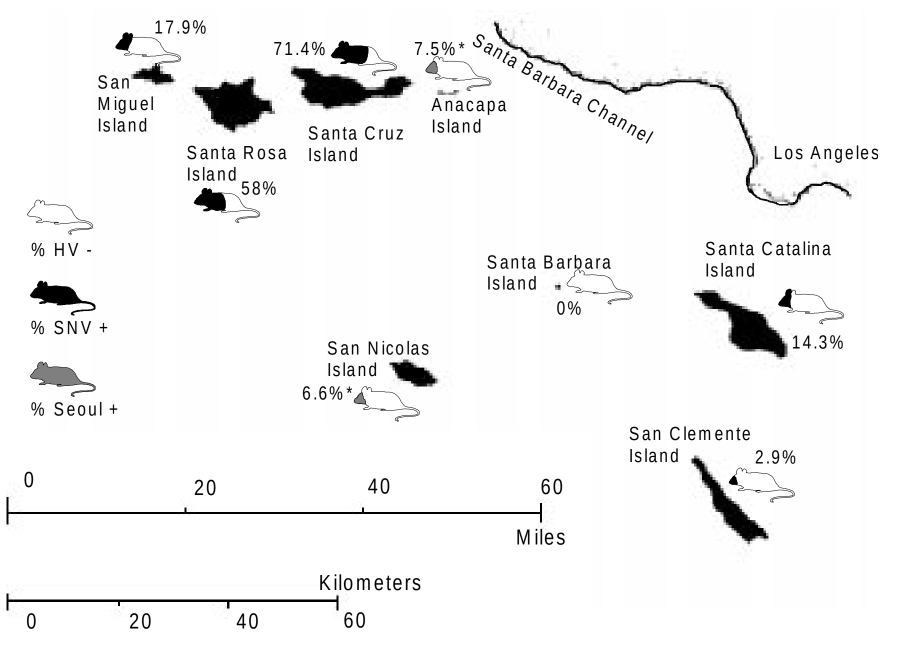

Figure 1. The California Channel Islands, with prevalence of Sin Nombre virus (SNV) and Seoul* hantaviruses on each island in 1994 (6). Channel Islands National Park comprises San Miguel, Santa Rosa, Santa Cruz, Anacapa, and Santa Barbara Islands.

others living or working on the islands in close contact with mice were also tested.

These tests indicated that the prevalence of SNV antibodies was 0% to 71% in mice tested from each island and that cross-reactivity to the Seoul hantavirus occurred on some islands (6) (Figure 1). Prevalence values for Santa Cruz (71%) and Santa Rosa (58%) Islands were higher than for any mainland population (1). No antibodies to hantaviruses were found in blood from any Channel Islands National Park or Santa Rosa ranch employees, including those living in mouse-infested cabins on islands with high hantavirus antibody prevalence in mice (K. Reilly, pers. comm.). Despite the lack of evidence of previous infection, concern for the health of visitors and employees remains.

*P. maniculatus* populations were sampled at only one location on each island during the 1994 survey. To understand the dynamics of the *P. maniculatus*–SNV relationship, the geographic variability of SNV on each island should be determined, requiring a more extensive sampling program. Few surveys have estimated rodent population densities in conjunction with testing for hantavirus; thus, the actual percentage of a population carrying the virus cannot be estimated, and prevalence values between sites cannot be compared (7,8). Furthermore, because mice were not trapped systematically and mouse densities were not estimated during sampling in 1994, comparisons between islands or sample times must be made with caution. Simultaneous monitoring of mouse populations and SNV prevalence in them should improve understanding of viral transmission from mouse to mouse and of the dynamics of changes in SNV antibody prevalence relative to mouse population fluctuations. The objectives of this pilot study were to determine whether prevalence differs spatially among populations on each island and between islands and whether prevalence differs temporally within each population.

We sampled mice at two locations on San Miguel, Santa Rosa, and Santa Cruz Islands. Sites were far enough apart that individual mice were unlikely to move directly from one site to the other. One site on each island was near concentrated human activity; the sites included the areas sampled in 1994 but not the exact locations. Mice were trapped in September 1995 (San Miguel and Santa Rosa Islands) and in February (San Miguel Island) 1996 and March (Santa Rosa and Santa Cruz Islands) 1996. These months generally represent the high and low population levels in the annual cycle on the islands (5). We trapped three nights at each site using 200 Sherman live traps arranged in a radial web design (9). Mouse population densities were estimated directly by the distance sampling theory (10). The number of new mice captured each day in each ring of traps is used to calculate density (number of mice per hectare) with the program DISTANCE (10).

Blood was collected from the suborbital sinuses of all mice captured except those from the San Miguel Island airstrip web in September 1995; only 34 of 247 mice captured there were sampled because of time constraints. Blood was refrigerated until transfer to the University of California, Davis. An enzyme immunosorbent assay with recombinant antigen (1) was used to determine the percentage of infected rodents and their antibody titer. Samples collected in September 1995 were analyzed by the Centers for Disease Control and Prevention; samples from February and March 1996 were analyzed at the University of California, Davis.

On the three islands, 531 mice were captured in 6,000 trap nights. Of these, 316 were tested for SNV antibodies, and 54 were positive (overall prevalence estimate 17%). Capture success was lower in 1996 on all islands as were density estimates (Table). We caught no mice at one web on Santa Rosa Island in September 1995, and only one mouse, which was seropositive, on the other web; a few days earlier on San Miguel Island we had caught 365 mice in the two webs. More mice were caught in September at the San Miguel Island airstrip web than at any other site or sample period (Table), but the program DISTANCE generated an unrealistic density estimate (> 8,000 mice per hectare). The number of mice caught near the center of the web was apparently too large to fit the models in DISTANCE (D. Anderson, pers. comm.). Therefore, we generated a naive density estimate for the San Miguel Island airstrip population in 1995 by dividing the total number of mice caught by the area covered by the web. We caught nine mice on Santa Cruz Island, two *P. maniculatus* on the Ranch web, and six *P. maniculatus* and one *R. megalotis* on the East Val web. Density at the East Val web was estimated as total mouse density using all seven mice. The density of *P. maniculatus* (Table) was calculated by multiplying total density by

Table. Numbers of *Peromyscus maniculatus* captured and tested on San Miguel (SMI), Santa Rosa (SRI), and Santa Cruz (SCI) Islands in 1995 and 1996, and results of serologic assay on blood collected from captured mice

| Island (Site) | Dates | Total captures (no.) | Mice (no.) | Density no. $ha^{-1}$ (SE) | Tested for SNV (no.) | SNV-positive (%) | Female: Male | Female: Male SNV-positive |
|---|---|---|---|---|---|---|---|---|
| SMI (Helipad) | 3-5 Sep 95 | 152 | 118 | 104.3 (9.6) | 116 | 15.5 | 59:57 | 6:12 |
| SMI (Helipad) | 15-17 Feb 96 | 67 | 49 | 43.3 (6.19) | 49 | 14.3 | 25:24 | 3:4 |
| SMI (Airstrip) | 3-5 Sep 95 | 353 | 247 | 218[a] (NA) | 34 | 5.9 | 17:17 | 1:1 |
| SMI (Airstrip) | 15-17 Feb 96 | 90 | 70 | 61.9 (7.4) | 70 | 30 | 37:33 | 10:11 |
| SRI (Airstrip) | 7-9 Sep 95 | 2 | 1 | NA | 1 | 100 | 0:1 | 0:1 |
| SRI (Torrey) | 7-9 Sep 95 | 0 | 0 | NA | 0 | NA | 0:0 | 0:0 |
| SRI (Campgr) | 27-29 Mar 96 | 43 | 22 | 19.5 (4.15) | 22 | 13.6 | 9:13 | 0:3 |
| SRI (Cherry) | 27-29 Mar 96 | 27 | 15 | 13.3 (3.42) | 15 | 13.3 | 5:10 | 0:2 |
| SCI (East Val) | 23-25 Mar 96 | 10 | 6 | 5.3 (2.01) | 6 | 0.0 | 2:4 | 0:0 |
| SCI (Ranch) | 23-25 Mar 96 | 5 | 2 | NA | 2 | 0.0 | 1:1 | 0:0 |

[a] Density was not estimated using DISTANCE, but directly, as the number of mice caught (247) divided by the area covered by the web (1.13 ha) .

the proportion of *P. maniculatus* (0.857) caught. The eight *P. maniculatus* and the *R. megalotis* were seronegative for SNV antibodies.

The prevalence of SNV is plotted against estimated mouse density for all island sampling sites and periods (Figure 2). The prevalence of SNV was similar for the San Miguel Island helipad population in both sampling periods and for the two Santa Rosa Island populations sampled in March 1996, despite densities ranging from 13 to 104 mice per hectare (Figure 2). The lowest prevalence occurred in the densest population, and the highest prevalence, found in the same population, occurred at a density similar to that of other locations (Figure 2). These results indicate that infection rates may be independent of mouse population dynamics. Monitoring mouse population size may not be an effective predictor of infection, but it may still be the best monitoring tool because it indicates the likelihood of exposure to deer mice and their feces and urine and thus the potential risk for SNV.

Chi-square analysis of the proportions of seropositive female and male mice was done for San Miguel Island populations (each site for each sampling period, total numbers tested in 1995 and in 1996, and all mice tested from San Miguel Island during the study). All mice tested from Santa Rosa Island were also analyzed for differences in infection rates by sex; no differences were found.

In 1996, we caught only adult mice, but in September 1995, we caught juvenile, subadult, and adult mice on San Miguel Island. Significantly more adult mice (16/39) were seropositive than expected, and fewer juvenile (2/38) and subadult (0/39) were seropositive at the helipad web (chi-square = 29.6, $p < 0.0001$), and for all mice (juvenile: 2/52, subadult: 1/54, adult: 17/44) tested on San Miguel Island (chi-square = 34.6, $p < 0.0001$). Douglass et al. (8) found a similar

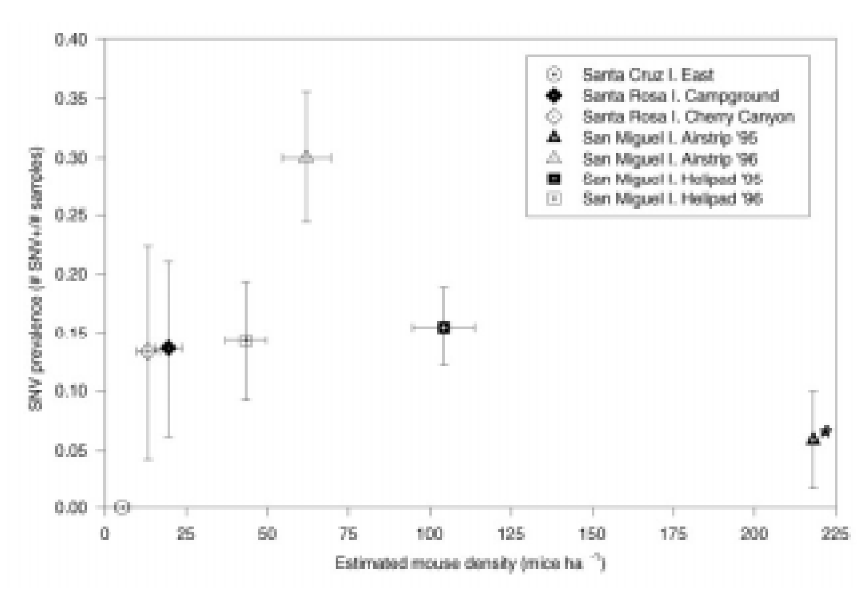

Figure 2. Deer mouse densities (±SE) vs Sin Nombre virus (SNV) prevalence (±SE) for three Channel Islands.*Density estimated directly as total number of mice caught per area of web.

pattern in Montana populations, but Jay et al. (6) did not find age differences in California mice.

We tried to trap near previous sampling locations, but only the San Miguel Island helipad site overlapped an area sampled in 1994. SNV prevalence was roughly the same at this site for our two sampling dates (16% and 14%), despite a large decline in numbers of mice. Both estimates were similar to the prevalence (18%) found in January 1994.

Our data indicate that the prevalence of SNV in mouse populations is different on different islands and that various locations on a single island may have specific dynamics. For example, density declined in both the helipad and airstrip populations from September 1995 to February 1996; SNV prevalence remained essentially the same in the helipad population but increased from 6% to 30% in the airstrip population (Figure 2). Prevalence estimates from our work were generally different from earlier testing, which implies at least spatial variation and perhaps temporal changes. Deer mice on the islands should be tested for SNV prevalence throughout the annual population cycle to determine how SNV is maintained in populations and how it is transferred from mouse to mouse.

### Acknowledgments

We thank Cathy Schwemm, Tim Coonan, and Greg Austin for valuable assistance with planning and logistics throughout the project; Jim Mills, Jamie Childs, and Pierre Rollin for advice and serology on the 1995 San Miguel Island blood samples; Jerry Johnson for his assistance with the funding process; Ken Yamada and Michele Jay for advice and safety equipment; Michele Jay for fieldwork; students from UC Davis, UC Santa Barbara, and Prescott College for their assistance with fieldwork; NPS and U.S. Navy boat crews for transportation; and NPS and Santa Cruz Island reserve staff for help on the islands. Funding from CDC was provided through the National Park Service.

**Tim B. Graham* and Bruno B. Chomel†**
*USGS, Biological Resources Division, Moab, Utah, USA; †University of California, Davis, California, USA

### References

1. Childs J, Ksiazek T, Spiropoulou C, Krebs J, Morzunov S, Maupin G, et al. Serologic and genetic identification of *Peromyscus maniculatus* as the primary rodent reservoir for a new hantavirus in the southwestern United States. J Infect Dis 1994;169:1271-80.
2. Nichol S, Spiropoulou C, Morzunov S, Rollin P, Ksiazek T, Feldmann H, et al. Genetic identification of a hantavirus associated with an outbreak of acute respiratory illness. Science 1993;262:914-7.
3. Centers for Disease Control and Prevention. Hantavirus pulmonary syndrome—United States, 1995-1996. MMWR Morb Mortal Wkly Rep 1996;45:291-5.
4. Hjelle B, Chavez-Giles F, Torrez-Martinez N, Yamada T, Sarisky J, Ascher M, Jenison S. The dominant glycoprotein epitope of Four Corners hantavirus is conserved across a wide geographic area. J Gen Virol 1994;75:2881-8.
5. Drost C, Fellers G. Density cycles in an island population of deer mice, *Peromyscus maniculatus*. Oikos 1991;60:351-64.
6. Jay M, Ascher M, Chomel B, Madon M, Sesline D, Enge B, et al. Seroepidemiologic studies of hantavirus infection among wild rodents in California. Emerg Infect Dis 1997;3:183-90.
7. Parmenter R. Pecos National Historic Park mammal survey data help solve hantavirus mystery. Park Science 1995;15:12-3.
8. Douglass R, Van Horn R, Coffin K, Zanto S. Hantavirus in Montana deer mouse populations: preliminary results. J Wildl Dis 1996;32:527-30.
9. Anderson D, Burnham K, White J, Otis D. Density estimation of small-mammal populations using a trapping web and distance sampling methods. Ecology 1983;64:674-80.
10. Buckland S, Anderson D, Burnham K, Laake J. Distance sampling. New York: Chapman and Hall, 1993.
